# Supplementary material for: Identification of Cis-Regulatory Sequences Controlling Pollen-Specific Expression of Hydroxyproline-Rich Glycoprotein Genes in Arabidopsis thaliana
Source: Plants (Basel). 2020 Dec 10;9(12):1751. doi: 10.3390/plants9121751 (PMC7763877; doi:10.3390/plants9121751)
Supplement: Supplementary file 1 [file plants-09-01751-s001.pdf]

Supplementary Materials:

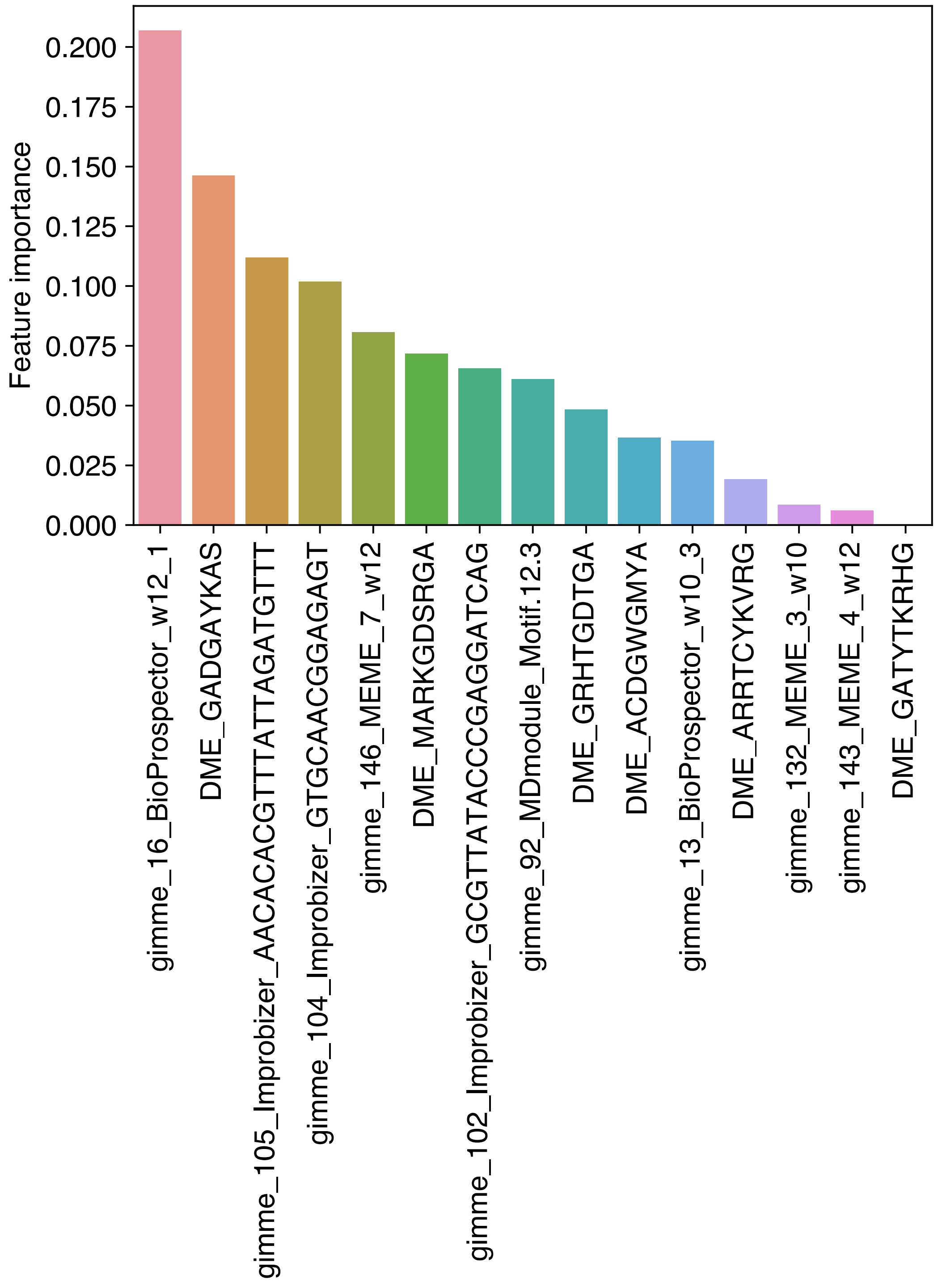

Figure S1. Feature importance bar plot of the 15 pollen-specific motifs. Feature importance was based on the gradient boosting tree model.
